# Bacterial Diversity, Structure, and Function in Rhizosphere and Bulk Soils of Grapevines: Comparing Gravelly, Calcareous, and Aeolian Sandy Textures

**DOI:** 10.3390/microorganisms14071504

**Published:** 2026-07-09

**Authors:** Haiwu Zheng, Yanxia Zhang, Zhenping Wang, Dongmei Li

**Affiliations:** 1 School of Life Sciences, Ningxia University, Yinchuan 750021, China; 1358649010@163.com; 2 Vocational and Technical College, Inner Mongolia Agricultural University, Baotou 014010, China; 3 Pomology Institute, Shanxi Agricultural University, Jinzhong 030600, China; 4 College of Enology and Horticulture, Ningxia University, Yinchuan 750021, China

**Keywords:** soil texture, bacterial community structure, rhizosphere–bulk gradient, shotgun metagenomics, vineyard terroir, alkaline ecosystem

## Abstract

Soil texture is a key determinant shaping bacterial communities in vineyard ecosystems, yet how different soil textures modulate bacterial characteristics in rhizosphere versus bulk soils during grapevine growth remains poorly understood. This study collected rhizosphere and bulk soil samples from five commercial *Vitis vinifera* cv. Cabernet Sauvignon vineyards in the eastern piedmont of Helan Mountain, Ningxia, China, spanning three distinct textures (gravelly, calcareous, and aeolian sandy soils). Shotgun metagenomic sequencing, soil physicochemical analysis, and four soil enzyme activity (alkaline phosphatase, urease, catalase, and invertase) measurements were conducted, using PERMANOVA and RDA to identify dominant driving factors. The results showed that bacteria accounted for 97.6% of all annotated sequences, representing the dominant group in soil microbial communities. Significant differences in bacterial abundance and alpha diversity (Chao1, ACE, Shannon, and Simpson) were observed in bulk soils across textures, whereas rhizosphere soils showed significant abundance differences but similar diversity levels. However, the 50 cm bulk soil sampling distance may have attenuated the true rhizosphere effect, and these findings should be interpreted with this methodological constraint in mind. Notably, bacterial community structure differed significantly between soils of the same pedogenic type but different textures, confirming that soil texture, rather than pedogenic classification, is the primary driver. Thirteen dominant bacterial phyla (>1% relative abundance) were identified, with Proteobacteria (47.7%), Actinobacteriota (22.9%), and Acidobacteriota (6.5%) as the main taxa. Mantel tests revealed significant correlations between nitrogen, phosphorus, organic matter contents and enzyme activities in rhizosphere soils (r ≥ 0.4, *p* < 0.01). RDA indicated that total phosphorus (TP), organic matter (OM), alkali-hydrolyzable nitrogen (AN), Mg, pH, available K (AK), and enzyme activities were key drivers of bacterial community structure (*p* < 0.05). Annotated metabolic functions based on KEGG orthology indicated lower overall metabolic pathway abundances in gravelly soils compared to calcareous and aeolian sandy soils. In conclusion, soil texture, rather than broad pedogenic classification, primarily shapes vineyard bacterial communities, providing a theoretical basis for precision viticulture and sustainable soil management.

## 1. Introduction

Grapevines (*Vitis vinifera* L.) are among the most widely cultivated fruit crops globally, with wine being one of the most economically important alcoholic beverages [1,2]. The French concept of “terroir” encompasses the complex interplay of vine type, physical environment, and human management practices that confer distinctive qualities to wines [3]. “Terroir” fundamentally influences grapevine growth, development, and berry quality, ultimately determining wine flavor profiles [3,4,5,6]. Even within the same cultivar and region, wines from different soil conditions exhibit significant quality variation, driving increasing research attention toward environmental factors and their underlying mechanisms [7,8].

Soil is one of the most critical components of “terroir”. Physical and chemical properties—including texture, structure, fertility, and moisture—directly govern root growth patterns and nutrient acquisition efficiency [9,10]. Soil texture, defined by the relative proportions of sand, silt, clay, and gravel particles, regulates water infiltration, aeration, and root penetration, thereby indirectly shaping microbial habitat heterogeneity [11,12,13]. Soil microorganisms serve as sensitive indicators of soil health, with physicochemical properties profoundly shaping microbial community characteristics [14,15].

Soil bacteria, fungi, and archaea perform essential ecosystem functions, including organic matter decomposition, nutrient cycling (C, N, and P), and soil structure formation [16,17,18]. Bacterial communities account for 70–90% of the soil microbial biomass [19]. Different textures create distinct microhabitats: clay particles offer protective niches and water retention, sand particles promote aeration but limit nutrient adsorption, and gravel content increases physical heterogeneity and drainage [20,21]. The rhizosphere, defined as the soil zone directly influenced by root exudates, represents a hotspot of microbial activity and diversity [22]. Plants release diverse compounds (ethylene, sugars, amino acids, organic acids, and polysaccharides) into the rhizosphere, selectively enriching specific microbial populations [23,24,25]. Recent metabolomic studies have revealed that root exudate composition varies with soil texture, particularly in coarse-textured soils where limited water retention constrains exudate diffusion [26]. In vineyards, root exudates from *V. vinifera* can recruit beneficial bacteria, enhancing nutrient acquisition and pathogen resistance [27,28]. However, the rhizosphere effect may vary with soil texture: coarse-textured soils with limited water retention may constrain exudate diffusion and microbial colonization [29].

The eastern foothills of the Helan Mountains in Ningxia, China, represent an emerging premium wine-producing region with diverse soil textures, including gravelly soils, aeolian sandy soils, and calcareous soils [30,31]. Previous studies in this region have primarily focused on fungal community variation [32] or soil improvement effects on microbial diversity [33], but systematic comparisons of bacterial communities across defined texture gradients remain limited. Furthermore, most studies have relied on amplicon sequencing (16S rRNA gene), which captures taxonomic composition but misses functional potential. Recent shotgun metagenomic studies in the Ningxia wine region have begun to address this gap [33], yet none have explicitly compared rhizosphere and bulk soils across fine-scale texture gradients. Shotgun metagenomic sequencing offers advantages by simultaneously resolving taxonomic and functional gene profiles, providing genomic context for abundant community members. Recent advances in strain-resolved metagenomics and long-read sequencing have further enhanced the ability to link community composition to functional potential in complex soil environments [34,35]. Based on the above literature, it was hypothesized that soil texture (gravel content, sand/silt/clay ratio) rather than broad pedogenic classification is the primary driver of bacterial community differentiation in vineyard soils; the rhizosphere effect (root exudate enrichment) homogenizes bacterial alpha diversity across different textures, but not community composition; and gravelly soils with higher organic matter support higher enzyme activities but lower annotated metabolic pathway diversity due to selective pressure from coarse texture. The objectives were to characterize bacterial communities in rhizosphere versus bulk soils across three distinct textures using shotgun metagenomics; identify key physicochemical and biological drivers through multivariate analysis; and link community structure to annotated metabolic functions.

## 2. Materials and Methods

### 2.1. Study Area Description

This study was conducted in the eastern foothills of the Helan Mountains in Ningxia, China (38°4′–38°57′ N, 105°50′–106°04′ E). The region has a temperate continental arid climate with 2850–3110 annual sunshine hours, mean annual temperature of 8.5–9.5 °C, and annual precipitation of 150–200 mm (concentrated in July–September). The active accumulated temperature (≥10 °C) ranges from 3100 to 3500 °C·d. The area is globally recognized as an emerging premium wine grape-producing region.

**Figure 1:**
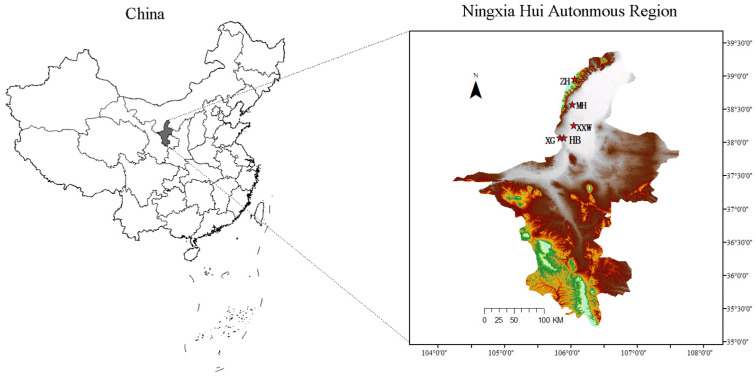
A map showing the location of the study site.

In 2021, five commercial vineyards were selected representing three distinct soil textures (Figure 1; Table S1):

Gravelly sandy loam soils:

Zhihuiyuanshi vineyard (ZH): N 38°57′98.35″, E 106°04′23.47″; 49.35% gravel (>2 mm), 47.16% sand, 2.04% silt, 1.45% clay; bulk density 1.51 g/cm^3^, specific gravity 2.68, porosity 42%;

Meihe vineyard (MH): N 38°34′24″, E 106°01′39″; 54.51% gravel, 41.48% sand, 2.56% silt, 1.46% clay; bulk density 1.38 g/cm^3^, specific gravity 2.70, porosity 49%;

Calcareous clay-loam soils:

Xige vineyard (XG): N 38°4′37″, E 105°50′36″; 9.92% gravel, 66.55% sand, 13.69% silt, 9.85% clay; bulk density 1.45 g/cm^3^, specific gravity 2.52, porosity 44%; CaCO_3_ content 8.7%;

Hongbao vineyard (HB): N 38°4′23″, E 105°53′54″; 11.07% gravel, 62.76% sand, 15.38% silt, 10.79% clay; bulk density 1.44 g/cm^3^, specific gravity 2.41, porosity 40%; CaCO_3_ content 7.2%;

Aeolian sandy soil:

Xixiawang vineyard (XXW): N 38°15′40″, E 106°2′41″; 3.87% gravel, 79.50% sand, 9.26% silt, 7.37% clay; bulk density 1.68 g/cm^3^, specific gravity 2.70, porosity 38%.

On HB classification, although HB showed lower total Ca concentration (12.4 g/kg) compared to XXW (28.6 g/kg) (Figure S2), its CaCO_3_ content (7.2%), clay content (10.79%), and bulk density (1.44 g/cm^3^) confirmed calcareous clay-loam classification. The lower total Ca concentration reflects higher leaching intensity at this site due to steeper slope position and higher porosity (40% vs. 38% in XXW). While the direct measurements of slope gradient or drainage rates are not available, the classification is supported by the co-occurrence of elevated CaCO_3_ (7.2%), clay content (10.79%), and bulk density (1.44 g/cm^3^), all consistent with calcareous clay-loam pedogenesis. Therefore, without explicit topographic or hydrological data, this classification relies on edaphic proxy variables and should be considered provisional.

All vineyards used *V. vinifera* cv. Cabernet Sauvignon on own-roots, trained to a modified vertical shoot positioning system with north–south row orientation, 1.0 m × 3.5 m planting spacing, 9–12 shoots and 17–21 clusters per meter, clusters positioned 0.3–1.2 m above ground. Vineyard age was 5–8 years. Yield was limited to 7500–9000 kg/ha. Drip irrigation (380 m^3^/ha per session) was applied monthly from May through August, with fertilizers and pesticides applied following regional winery guidelines uniformly across all sites. Specifically, all vineyards received identical basal fertilization (N-P-K compound fertilizer via drip fertigation) and standard sulfur-based fungicide applications for downy mildew control; no organic amendments or soil biocides were applied.

### 2.2. Sample Collection

Soils were sampled during the veraison period (E-L 35), a critical growth stage for microbial community establishment [36]. Sampling was conducted 7 days after the last irrigation event (380 m^3^/ha drip irrigation) to allow soil moisture to stabilize and minimize the transient disruptive effects of irrigation on microbial communities, while still capturing the moisture conditions representative of the vineyard’s routine management cycle. A stratified random sampling design was employed to capture within-vineyard heterogeneity.

In each vineyard, nine contiguous rows of uniform grapevine plants were pre-selected. Within these rows, three independent sampling points were established as technical replicates (*n* = 3 per vineyard per compartment), with each point consisting of three pooled subsamples (0.5 m radius) to reduce micro-scale variation. Each vineyard served as a biological replicate for its respective soil texture category. Total samples numbered 30 (5 vineyards × 2 compartments [rhizosphere/bulk] × 3 technical replicates).

At each sampling point, rhizosphere soil was collected from the root zone at 30–60 cm depth by gently brushing soil adhering to grapevine roots using a sterile brush [14]. Bulk soil (non-rhizosphere) was collected at the same depth from a point approximately 50 cm away from the trunk where no visible roots were present. This distance may not fully exclude fine root influence, representing a limitation.

Visible plant roots, debris, and rocks (>2 mm) were removed from each sample. Samples were placed in sterile plastic bags, transported on dry ice, and divided into two portions: one for physicochemical and enzyme analysis (stored at 4 °C, analyzed within 7 days) and one for DNA extraction (stored at −80 °C).

### 2.3. Soil Physicochemical and Biological Property Determination

Soil physicochemical properties were determined as follows. pH was measured using a Laici PHSJ-3F pH meter (Shanghai INESA Scientific Instrument Co., Ltd., Shanghai, China) in a 1:2.5 soil:water suspension. Electrical conductivity (EC) was measured using a Leici DDS-307A conductivity meter at 1:5 soil:water ratio (Shanghai INESA Scientific Instrument Co., Ltd., Shanghai, China). Total nitrogen (TN) was determined by the Kjeldahl method. Total phosphorus (TP) and available phosphorus (AP) were measured according to Chinese National Standards (GB 9837-88 [37] and NY/T 148-1990 [38]). Organic matter (OM) was determined by potassium dichromate oxidation (NY/T 1121.6-2006 [39]). Organic nitrogen was analyzed by diffusion methods [40]. Available potassium (AK) was determined by flame atomic absorption spectrometry [41]. Other mineral elements (Ca, Mg, Al, and Fe) were measured by inductively coupled plasma-atomic emission spectrometry (inductively coupled plasma-atomic emission spectrometry, ICP-AES; Optima 8000, PerkinElmer, Waltham, MA, USA).

Soil enzyme activities (biological properties) were determined by colorimetric methods as described by Song et al. [42]: alkaline phosphatase (ALP) by disodium phenyl phosphate hydrolysis at pH 8.5; urease (UA) by sodium phenolate colorimetry after urea incubation; catalase (Cat) by potassium permanganate titration after H_2_O_2_ decomposition; and invertase (IA) by 3,5-dinitrosalicylic acid colorimetry after sucrose hydrolysis.

All physicochemical and enzyme activity data are presented in Figure S2, with significant differences among sample groups indicated by different lowercase letters at the *p* < 0.05 level.

### 2.4. Soil DNA Extraction and Shotgun Metagenomic Sequencing

DNA extraction from the soil samples and subsequent library preparation for metagenomic sequencing were performed using the FastDNA™ SPIN Kit for Soil DNA Extraction (MP Biomedicals, Santa Ana, CA, USA). The genomic DNA concentration and quality were assessed using Nanodrop 2000 spectrophotometer (UV-Vis spectrophotometer, PerkinElmer Inc., Waltham, MA, USA). DNA integrity was further assessed by 1% agarose gel electrophoresis, and samples with OD260/280 ratio of 1.8–2.0 and concentration > 20 ng/μL were selected for library construction.

Qualified genomic DNA was fragmented to an average size of 400 bp (ranging from 200 bp to 600 bp) using a Covaris ME220 (Covaris LLC, Woburn, MA, USA). The fragmented DNA underwent the library construction step of end repair, dA-tailing and linker ligation steps, followed by the addition of Illumina adapters to both ends of the library DNA fragment using T4 DNA ligase (New England Biolabs, Ipswich, MA, USA). PCR amplification was performed to generate the final sequencing libraries, which were then subjected to quality control analyses. Libraries with insert size of 350–450 bp and concentration >2 nM were selected for sequencing.

Qualified libraries were then sequenced with the Illumina HiSeq platform (Illumina, Inc., San Diego, CA, USA) using a 2 × 150 bp paired-end sequencing mode to generate FastQ data. The raw sequencing data were checked for quality using FastQC v0.11.9 (Babraham Bioinformatics, Cambridge, UK; https://www.bioinformatics.babraham.ac.uk/projects/fastqc/, accessed on 15 March 2024). Reads with adapter contamination, low quality (Q < 20), or ambiguous bases (N > 5) were removed using Trimmomatic (v0.39).

The clean reads from each sample were assembled de novo into contigs with metaSPAdes v3.11.1 (k-mer sizes: 21, 33, 55, 77). The clean reads were then aligned to the contigs using BWA-mem v0.7.17-r1188 (http://bio-bwa.sourceforge.net/, accessed on 15 March 2024). with default parameters. The assembly results were further filtered for redundancy using MMseqs2 v14-7e284 (https://github.com/soedinglab/MMseqs2, accessed on 15 March 2024, min-seq-id 0.95--cov-mode 1-c 0.9). Open reading frames (ORFs) were predicted using Prodigal (v2.6.3) with meta mode. The nucleotide sequences of non-redundant genes were aligned to the KEGG protein sequence database using DIAMOND v2.1.9 (https://github.com/bbuchfink/diamond, accessed on 15 March 2024, blastx--fast-e 1e-5). Taxonomic annotation was performed by aligning non-redundant contigs/genes to GTDB (Release 07-RS207) and NCBI RefSeq databases using MMseqs2 (-s 7). Functional genes were annotated against KEGG Orthology (KO) and mapped to KEGG pathways at levels 1–3.

Metabolic functions were categorized into 12 level-2 KEGG pathways: amino acid metabolism (AAM), carbohydrate metabolism (CM), energy metabolism (EM), metabolism of cofactors and vitamins (MCV), nucleotide metabolism (NM), lipid metabolism (LM), xenobiotics biodegradation and metabolism (XBM), metabolism of other amino acids (MOAA), biosynthesis of other secondary metabolites (BOSM), metabolism of terpenoids and polyketides (MTP), glycan biosynthesis and metabolism (GBM), and unclassified metabolism (UM).

The above mentioned procedures were conducted by Genesky Biotechnologies, located in Shanghai, China.

### 2.5. Statistical Analysis

For alpha diversity analysis, Chao1, ACE, Shannon, and Simpson indices were calculated based on the species abundance table (GTDB taxonomy at the species level) using the R package vegan (v2.6-4). Prior to diversity calculation, samples were rarefied to 90,000 reads per sample based on the species abundance matrix to ensure comparability. Good’s coverage was calculated as C = 1 − (n_1_/N), where n_1_ is the number of singleton species and N is the total number of individuals, to assess sampling completeness.

For beta diversity analysis, Bray–Curtis dissimilarity matrices were computed at the genus level using relative abundances. Principal coordinate analysis (PCoA) was performed to visualize community structure differences. PERMANOVA (Adonis2 function, vegan package v2.6-4 in R v4.3.1, 999 permutations) was used to test the effects of Texture (three levels: gravelly, calcareous, and aeolian sandy) and Compartment (two levels: rhizosphere and bulk) on community composition, including their interaction.

LEfSe analysis was conducted with stringent parameters: LDA score > 3.5 and Benjamini-Hochberg FDR correction (q < 0.05). Only taxa meeting both criteria were retained as significant biomarkers.

For univariate comparisons, one-way ANOVA followed by Tukey’s Honestly Significant Difference (HSD) post hoc test was used to compare alpha diversity indices and enzyme activities across the five vineyards (α = 0.05). All cross-site comparisons were interpreted with caution given the limited number of independent vineyards per texture category (two for gravelly, two for calcareous, and one for aeolian sandy) and the hierarchical nesting of technical replicates within vineyards. Different lowercase letters in each column indicate significant differences among sample groups at *p* < 0.05. Mann–Whitney U test was applied for pairwise rhizosphere vs. bulk comparisons within each vineyard, with significance levels indicated as * *p* < 0.05, ** *p* < 0.01, *** *p* < 0.001, **** *p* < 0.0001. Type I error inflation from multiple t-tests was avoided by using ANOVA with Tukey HSD for all cross-site comparisons.

For phylum-level comparisons across all sites, different uppercase letters indicate significant differences at *p* < 0.05 between the combined rhizosphere (R) and combined bulk (NR) groups.

For multivariate associations, Mantel tests (999 permutations) correlated Bray–Curtis community dissimilarity with Euclidean distance of environmental variables. Redundancy analysis (RDA) was performed using the vegan package (R v4.2.1) to identify environmental drivers of community structure. Forward selection (ordiR2step function) was applied to select significant constraints, with significance tested by permutation ANOVA (999 permutations). Variance inflation factors (VIF) were checked to ensure collinearity among environmental variables was acceptable (VIF < 10). Given 16 initial explanatory variables and 30 samples, the final model (11 constraints, R^2^adj = 0.62) may be overparameterized; therefore, TP (R^2^ = 0.585), OM (R^2^ = 0.518), and AN (R^2^ = 0.467) are interpreted as primary drivers, while variables with R^2^ < 0.30 are treated as secondary or potentially spurious.

For correlation analysis, Pearson correlation coefficients were calculated between alpha diversity indices and individual environmental variables. All continuous variables were log-transformed when necessary to meet normality assumptions.

For metabolic function analysis, functional genes were annotated against KEGG pathways. Relative abundances of level-2 pathways were compared by Kruskal–Wallis test followed by Dunn’s post hoc test. Different lowercase letters indicate significant differences among sample groups at *p* < 0.05, and different uppercase letters indicate significant differences between combined R and NR groups at *p* < 0.05. Annotated metabolic functions based on KEGG orthology represent genomic potential inferred from gene presence, not direct metabolic measurements. All statistical analyses were conducted in R v4.2.1 (vegan, ggplot2, dplyr packages) and SPSS 26.0.

## 3. Results

### 3.1. Soil Physicochemical Properties and Enzyme Activities

Soil physicochemical properties varied significantly across the five vineyards (Figure S2; Table S1). Gravelly soils (ZH, MH) exhibited significantly higher organic matter (OM: ZH 1.82%, MH 1.76%) and total phosphorus (TP: ZH 0.89 g/kg, MH 0.85 g/kg) compared to aeolian sandy soil (XXW: OM 0.68%, TP 0.42 g/kg) and calcareous soils (XG: OM 1.45%, TP 0.72 g/kg; HB: OM 1.38%, TP 0.68 g/kg) (ANOVA, *p* < 0.05). All soils were alkaline (pH 8.21–8.67), with HB showing the lowest pH (8.21) despite calcareous classification, likely due to localized organic acid accumulation and higher leaching intensity. Calcium content, expressed on a soil mass basis, ranged from 12.4 g/kg (HB) to 28.6 g/kg (XXW). Bulk density ranged from 1.38 g/cm^3^ (MH) to 1.68 g/cm^3^ (XXW) (Table S1).

Enzyme activities exhibited texture-dependent patterns (Figure S2). ALP, UA, Cat, and IA were highest in gravelly soils (ZH, MH) and lowest in aeolian sandy soil (XXW) (Tukey HSD, *p* < 0.05), consistent with higher OM and nutrient contents in gravelly soils. Different lowercase letters in Figure S2 indicate significant differences among sample groups at the *p* < 0.05 level. Notably, enzyme activities were biological properties responding to substrate availability, distinct from abiotic physicochemical parameters.

### 3.2. Bacterial Abundance and Diversity Across Soil Textures

Shotgun metagenomic sequencing generated 6.2–8.7 Gb of raw data per sample. After quality filtering (FastQC), high-quality reads were assembled and annotated against GTDB and NCBI RefSeq databases. Taxonomic annotation was performed by aligning non-redundant contigs to the GTDB (Release 07-RS207) and NCBI RefSeq databases using MMseqs2 (-s 7), which yielded 930,247 bacterial sequences, accounting for 97.6% of total aligned reads. Gene-level clustering was performed using MMseqs2 (min-seq-id 0.95--cov-mode 1-c 0.9) to generate non-redundant gene catalogs for downstream functional annotation. Rarefaction curves reached asymptote for all samples (Figure S1), and Good’s coverage exceeded 98% (Table 1), confirming adequate sequencing depth.

Alpha diversity was analyzed using Chao1, ACE, Shannon, and Simpson indices (Table 1). One-way ANOVA followed by Tukey’s HSD test revealed that Chao1 and ACE were significantly higher in rhizosphere soil of HB (R_HB: Chao1 6517, ACE 6534) compared to MH, XXW, ZH, and XG (*p* < 0.05). Similarly, NR_HB (Chao1 6284, ACE 6351) showed higher alpha diversity than XG (Chao1 5686, ACE 5698) and ZH (Chao1 5196, ACE 5266). The Simpson index did not differ significantly among rhizosphere soils, but NR_ZH (0.0228) was significantly higher (lower diversity) than MH (0.0168), XXW (0.0143), and HB (0.0138).

These results suggest that soil texture significantly influenced bacterial abundance and diversity in bulk soils, whereas in rhizosphere soils, texture-dependent diversity differences were less pronounced, likely due to homogenizing effects of root exudates. Notably, within the same texture class, XG and HB (both calcareous) showed significant Chao1 differences between rhizosphere (5946 vs. 6517) and bulk soils (5686 vs. 6284), suggesting that fine-scale differences in soil properties (including but not limited to clay content: 9.85% vs. 10.79%) may contribute to community divergence within broad texture classes. However, given the small magnitude of these differences and the presence of uncontrolled confounding factors (e.g., slope position, microclimate, and localized organic matter distribution), causal attribution to clay content alone is not warranted. The observed differences between rhizosphere and bulk soils within the same vineyard likely reflect root-induced changes in soil structure (aggregate formation, organic matter enrichment, and localized nutrient redistribution) rather than inherent particle size variation.

### 3.3. Bacterial Community Composition and Structure

**Figure 2:**
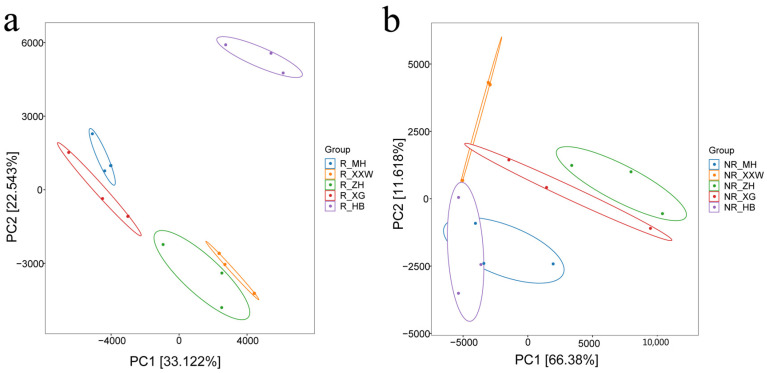
PCoA analysis of microbial community based on Bray–Curtis distance; the points of different colors represent sample groups from different environment conditions: (**a**) bacterial community in rhizosphere soils and (**b**) bacterial community in bulk soils.

Principal coordinate analysis (PCoA) based on Bray–Curtis dissimilarity at the genus level revealed clear separation among the five vineyards in rhizosphere soils (Figure 2a), indicating distinct bacterial community structures across textures. PERMANOVA confirmed the significant effects of Texture (R^2^ = 0.28, *p* = 0.001) and Compartment (R^2^ = 0.15, *p* = 0.003), with a non-significant interaction (R^2^ = 0.08, *p* = 0.12).

In bulk soils (Figure 2b), XXW and XG, XXW and HB, and MH and HB showed overlap, while XG and HB (both calcareous) were distinctly separated, as were ZH and MH (both gravelly). Thus, within the same pedogenic type, texture differences (gravel content: 9.92% vs. 11.07%; clay: 9.85% vs. 10.79%) exerted stronger effects on community structure than broad soil classification.

**Figure 3:**
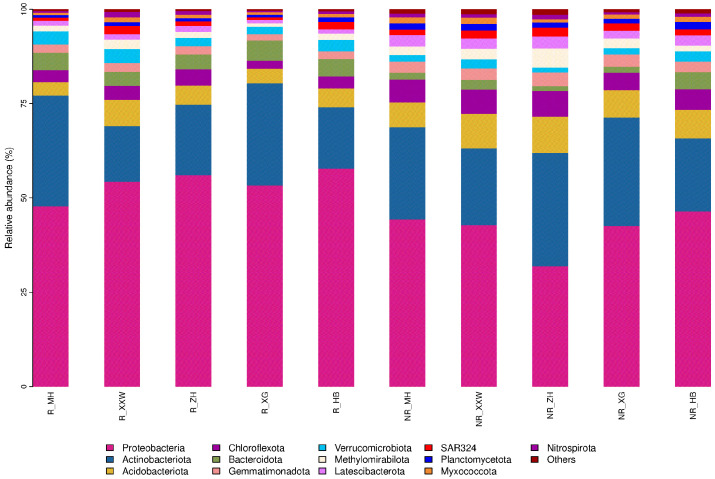
Histogram of phylum horizontal bacterial community structure analysis.

Thirteen bacterial phyla with relative abundances > 1% were identified (Figure 3; Table S2). The dominant phyla were Proteobacteria (47.7%), Actinobacteriota (22.9%), Acidobacteriota (6.5%), Chloroflexota (4.6%), Bacteroidota (3.4%), Gemmatimonadota (2.6%), Verrucomicrobiota (2.4%), Methylomirabilota (2.2%), Latescibacterota (2.0%), SAR324 (1.6%), Planctomycetota (1.2%), Myxococcota (1.1%), and Nitrospirota (0.8%) (Table S2).

Mann–Whitney U test revealed that 11 of 13 phyla (except Actinobacteriota and Nitrospirota) showed highly significant differences between rhizosphere and bulk soils (Table S2: **** *p* < 0.0001). Proteobacteria was significantly more abundant in combined rhizosphere (R: 53.9%) than combined bulk (NR: 41.6%) (*p* < 0.05, uppercase letters). Conversely, Acidobacteriota (R: 4.9% vs. NR: 8.0%), Chloroflexota (R: 4.4% vs. NR: 5.9%), and Latescibacterota (R: 1.4% vs. NR: 2.0%) were significantly higher in bulk soils (**** *p* < 0.0001).

In different soil habitats, core bacteria abundance varied between rhizosphere and bulk soils. For example, Proteobacteria abundance was significantly lower in NR_ZH (31.8%) than in other bulk soils, while in rhizosphere soil, it was significantly higher in R_ZH (56.1%) and R_HB (57.8%) than in R_XXW (54.3%). SAR324 showed distinct patterns: in MH, ZH, and XG, its abundance was significantly lower in rhizosphere than in bulk soils, while in HB, R_HB (2.0%) was significantly higher than NR_HB (1.6%) (Table S2). In vineyards with the same texture and different fine-scale composition, such as XG and HB (both calcareous), significant differences existed in Proteobacteria (53.3% vs. 57.8%), Actinobacteriota (27.1% vs. 16.2%), Acidobacteriota (3.9% vs. 5.0%), and Verrucomicrobiota (1.9% vs. 3.0%) in rhizosphere soil. In their bulk soil, significant differences were observed in Proteobacteria, Acidobacteriota, Methylomirabilota, SAR324, and Myxococcota. Similarly, in ZH and MH (both gravelly), significant differences existed in Actinobacteriota, Bacteroidota, Verrucomicrobiota, Latescibacterota, SAR324, and Planctomycetota in rhizosphere soil. In bulk soil, significant differences were observed in Actinobacteriota, Bacteroidota, Verrucomicrobiota, Latescibacterota, SAR324, and Planctomycetota (Table S2).

Hence, the composition of bacterial communities in rhizosphere and bulk soils of grapevines varies significantly across different soil textures within the same broad soil type, challenging the notion that soil type (pedogenic classification) is the primary driver.

### 3.4. Discriminant Bacterial Taxa Across Habitats

**Figure 4:**
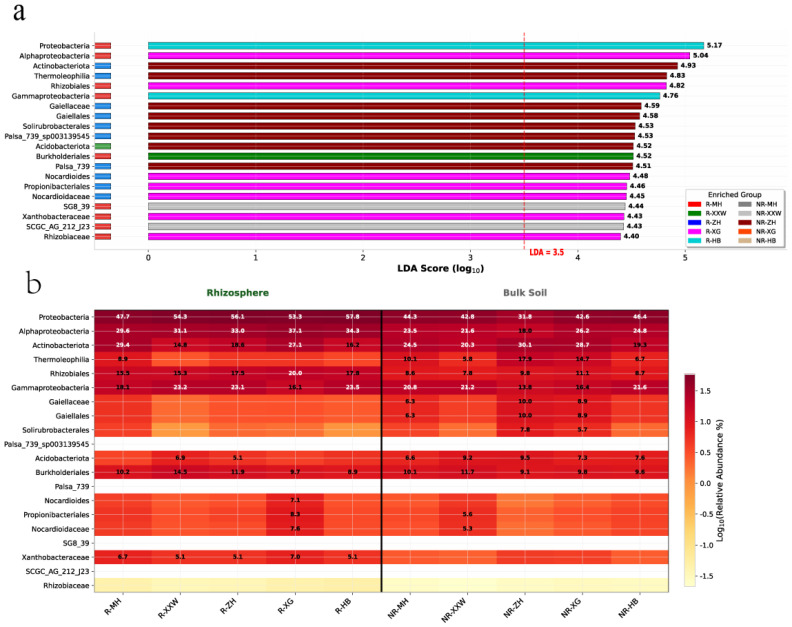
Linear discriminant analysis (LDA) effect size (LEfSe) of discriminant bacterial taxa across vineyard soil habitats. (**a**) Top 20 biomarkers (LDA > 3.5, Benjamini–Hochberg FDR q < 0.05) ranked by LDA score. Only five enriched habitats are represented: R-HB (cyan), R-XG (magenta), NR-ZH (dark red), R-XXW (green), and NR-XXW (light gray). The other five habitats (R-MH, R-ZH, NR-MH, NR-XG, and NR-HB) appear in other biomarkers listed in Supplementary Table S5 but not in the top 20. The red dashed line denotes the LDA = 3.5 threshold. (**b**) Relative abundance (%) of the top 20 biomarkers across all 10 habitat groups, with rhizosphere (left) and bulk soil (right) separated by a vertical black line. Values > 1% are shown in cells. Color intensity represents log10-transformed relative abundance.

Following stringent multiple-comparison correction (Benjamini–Hochberg FDR, q < 0.05) and an elevated LDA threshold (>3.5), LEfSe analysis identified 196 significantly discriminant bacterial taxa across habitats (Figure 4; Table S5). The top 20 biomarkers (LDA > 3.5, ranked by effect size) are presented in Figure 4a, comprising taxa primarily from *Proteobacteria* (44.4%), *Actinobacteriota* (21.4%), and *Acidobacteriota* (5.6%), with distinct enrichment patterns across textures and compartments. Notably, NR-ZH (gravelly bulk soil) harbored the highest number of biomarkers (n = 55, 28.1%), dominated by *Actinobacteriota*, *Chloroflexota*, and *Acidobacteriota*, while R-XG (calcareous rhizosphere) contained 46 biomarkers (23.5%), primarily from *Proteobacteria*. The complete list of 196 biomarkers, including taxonomic affiliation, LDA scores, *p*-values, and FDR q-values, is provided in Supplementary Table S5.

### 3.5. Correlations Between Enzyme Activities and Soil Properties in Rhizosphere Soils

**Figure 5:**
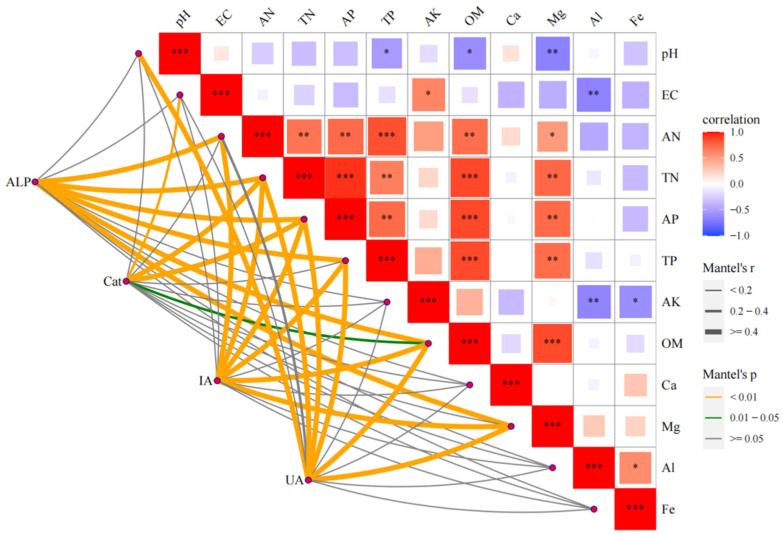
Mantel test of the correlation between enzyme activities and physicochemical properties in the rhizosphere soils. * *p* < 0.05, ** *p* < 0.01, *** *p* < 0.001. OM: the concentrations of soil organic matter; pH: soil pH; EC: soil electrical conductivity; AN: soil alkali-hydrolyzed nitrogen concentration; TN: soil total nitrogen concentration; AP: soil available phosphorus content; TP: soil total phosphorus concentration; AK: soil available K concentration; Ca: soil Ca concentration; Mg: soil Mg concentration; Al: soil Al concentration; Fe: Fe concentration in soil; ALP: soil alkaline phosphatase activity; UA: urease activity in soil; Cat: catalase activity in soil; IA: soil invertase activity.

Mantel tests revealed significant correlations between enzyme activities and soil properties in rhizosphere soils (Figure 5; *p* < 0.05). ALP showed significant correlations with AN, TN, AP, TP, OM, and Mg (r ≥ 0.4, *p* < 0.01). Cat correlated with EC (r ≥ 0.2, *p* < 0.01), TN (r ≥ 0.4, *p* < 0.01), AP (r ≥ 0.4, *p* < 0.01), and OM (r ≥ 0.2, *p* < 0.05). IA correlated with AN, TN, AP, TP, OM, and Mg (r ≥ 0.4, *p* < 0.01). UA correlated with pH, TN, AP, TP, OM, and Mg (r ≥ 0.4, *p* < 0.01).

These results indicate that nitrogen, phosphorus, and organic matter availability are key drivers of rhizosphere enzyme activity. The correlation between enzyme activities and OM content (except catalase) is consistent with the role of organic matter as the primary growth substrate for microorganisms.

### 3.6. Environmental Drivers of Bacterial Community Structure

**Figure 6:**
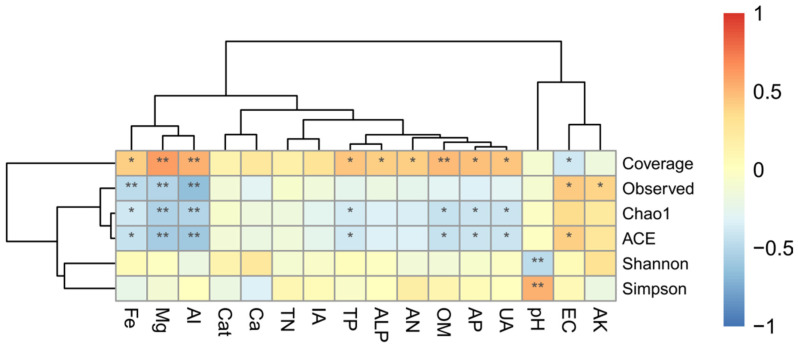
Correlation analysis between soil physicochemical properties and bacterial diversity. * *p* < 0.05, ** *p* < 0.01.

Pearson correlation analysis revealed that pH was negatively correlated with bacterial Shannon diversity (r = −0.42, *p* = 0.018). Al, Mg, Fe, TP, OM, AP, and UA were negatively correlated with Chao1 index (Figure 6).

Redundancy analysis (RDA) was performed using the 12 dominant bacterial phyla (cumulative relative abundance > 98%) as response variables and 16 soil properties as explanatory variables. Forward selection identified TP, OM, AN, Mg, pH, AK, UA, ALP, TN, IA, and AP as significant constraints (Table S3: permutation ANOVA, *p* < 0.05), while Ca, Fe, Al, Cat, and EC were non-significant (*p* > 0.05).

TP showed the strongest effect (RDA1: −0.046, RDA2: −0.999, r^2^ = 0.585, *p* = 0.001), followed by OM (r^2^ = 0.518, *p* = 0.001), AN (r^2^ = 0.467, *p* = 0.001), Mg (r^2^ = 0.399, *p* = 0.001), pH (r^2^ = 0.378, *p* = 0.002), AK (r^2^ = 0.319, *p* = 0.009), UA (r^2^ = 0.270, *p* = 0.016), ALP (r^2^ = 0.268, *p* = 0.019), TN (r^2^ = 0.235, *p* = 0.026), IA (r^2^ = 0.217, *p* = 0.032), and AP (r^2^ = 0.215, *p* = 0.036) (Table S3). Ca (r^2^ = 0.123, *p* = 0.156), Fe (r^2^ = 0.091, *p* = 0.286), Al (r^2^ = 0.035, *p* = 0.603), Cat (r^2^ = 0.032, *p* = 0.618), and EC (r^2^ = 0.014, *p* = 0.823) showed no significant effects.

**Figure 7:**
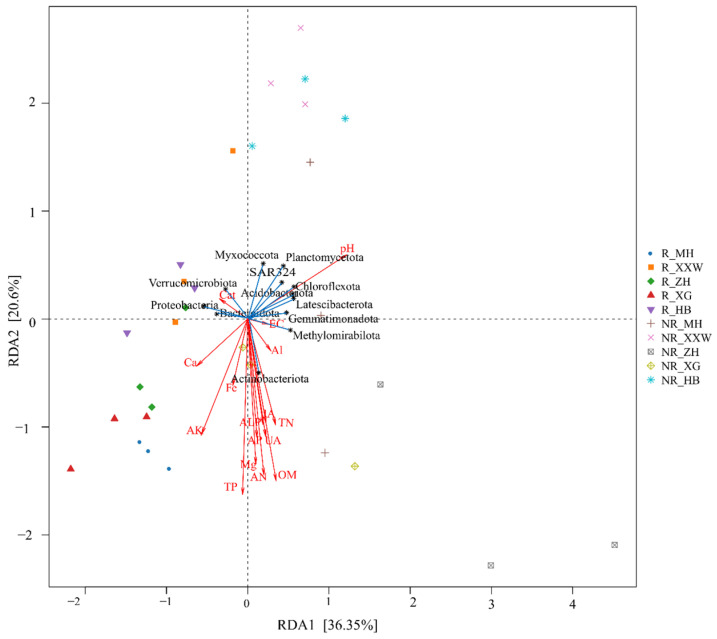
Redundancy analysis (RDA) was conducted between the 12 dominant bacterial phyla (*) (collectively comprising over 98% relative abundance) and 16 soil physicochemical properties.

Most phyla showed positive correlations with pH, except Actinobacteriota, Bacteroidota, Proteobacteria, and Verrucomicrobiota. AK, TN, TP, OM, ALP, AP, IA, and UA were negatively correlated with most phyla except Actinobacteriota. In particular, AK was negatively correlated with Bacteroidota and Proteobacteria, IA was negatively correlated with Methylomirabilota and Gemmatimonadota. TN, TP, OM, ALP, AP, AK, and UA were positively correlated with Methylomirabilota (Figure 7).

In RDA analysis, the influence of environmental parameters on biological variables is represented by arrows, where the length of the arrow is proportional to its importance. The angle between influencing factors represents their correlation: acute angles indicate a positive correlation, obtuse angles indicate a negative correlation, and right angles indicate no correlation between the two factors. Different shapes were used to distinguish rhizosphere (circles) and bulk (squares) soils, and colors to indicate soil textures (green = gravelly, brown = calcareous, and yellow = aeolian sandy).

### 3.7. Metabolic Functions of Rhizosphere and Bulk Soil Microbiomes

**Figure 8:**
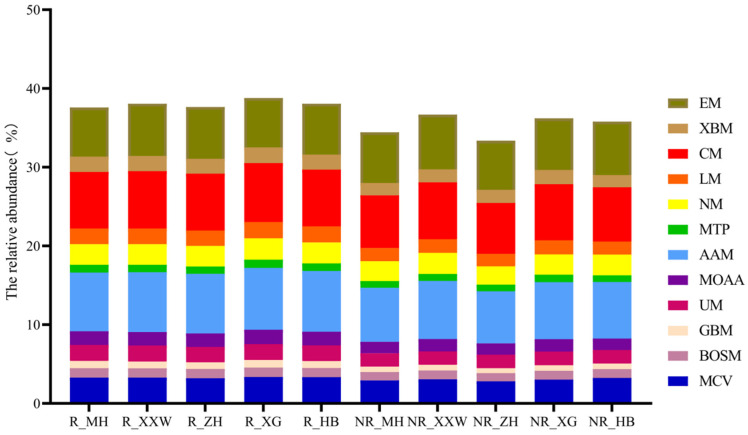
Metabolic function analysis of rhizosphere and bulk microbial communities in different grapevine soil habitats.

Metagenomic functional annotation against KEGG pathways revealed that amino acid metabolism (AAM) was the most abundant function across all samples (7.35%), followed by carbohydrate metabolism (CM: 7.08%), energy metabolism (EM: 6.51%), metabolism of cofactors and vitamins (MCV: 3.15%), and nucleotide metabolism (NM: 2.60%) (Figure 8; Table S4). Other detected pathways included lipid metabolism (LM: 1.85%), xenobiotics biodegradation (XBM: 1.79%), metabolism of other amino acids (MOAA: 1.62%), biosynthesis of secondary metabolites (BOSM: 1.14%), metabolism of terpenoids and polyketides (MTP: 0.92%), glycan biosynthesis (GBM: 0.80%), and unclassified metabolism (UM: 1.85%).

In both rhizosphere and bulk soils, ZH and MH (gravelly) showed consistently lower overall metabolic abundances than XG, HB (calcareous), and XXW (aeolian sandy). Unclassified metabolism (UM) showed no significant differences in bulk soils (Dunn’s test, *p* > 0.05), but CM, XBM, BSM, UM, MCV, and NM differed among bulk soil habitats. In rhizosphere soils, CM, XBM, BSM, UM, MCV, and NM showed no significant differences among textures, while AAM, EM, LM, and MTP differed significantly (*p* < 0.05).

Notably, MTP abundance was significantly higher in R_XG (1.04%) than in other rhizosphere and bulk soils (Table S4), potentially reflecting enhanced secondary metabolite production in calcareous clay-loam rhizospheres. UM abundance was lower in R_XG (1.96%) than in other rhizosphere soils.

Overall, annotated metabolic levels in gravelly soils were lower than in calcareous and aeolian sandy soils. Within the same habitat, all metabolism-related genes except EM were significantly more abundant in rhizosphere than bulk soils (Mann–Whitney, *p* < 0.05), consistent with root exudate-driven metabolic stimulation. These metabolic functions represent genomic potential based on gene presence, not direct metabolic measurements.

## 4. Discussion

### 4.1. Soil Texture Effects on Rhizosphere Bacterial Alpha Diversity

The interactions among soil texture, plant genotype, and growth stage exert complex effects on rhizosphere microbial communities. While some studies suggest soil properties override plant species effects [43], others demonstrate stronger plant influence [44]. Plants release diverse root exudates (ethylene, sugars, amino acids, organic acids, and polysaccharides) that selectively enrich specific microbial populations [25,45].

In this study, bulk soils showed significant texture-dependent diversity differences, whereas rhizosphere soils showed significantly less pronounced texture-dependent diversity differences compared to bulk soils (Shannon index range: 5.77–5.95 vs. 5.24–5.69 in bulk soils), suggesting that the rhizosphere effect may partially buffer texture-driven diversity variation. Specifically, R_HB showed the highest Chao1 (6517) and ACE (6534) among all rhizosphere soils, while NR_HB (Chao1 6284) also exceeded most bulk soils. We hypothesize that root exudates in the rhizosphere homogenized diversity across textures by providing a common carbon source, while texture-driven differences in nutrient availability (particularly P and N) modulated total abundance.

The negative correlation between pH and the Shannon diversity index (r = −0.42, *p* = 0.018) is specific to the narrow, highly alkaline range observed in this study (pH 8.21–8.67) and should not be interpreted as a universal rule. In acidic soils, the opposite trend often occurs, with diversity increasing as pH rises toward neutrality [46]. Within the studied alkaline range, this correlation likely reflects the dominance of alkaliphilic Actinobacteriota (abundance > 20% at pH > 8.5) in high-pH soils, which may competitively exclude acidophilic groups (e.g., Acidobacteriota prefer pH < 6.0), reducing community evenness. This aligns with Wang et al. [47], who confirmed pH as a master variable in arid ecosystems, with diversity peaking at intermediate pH (6.5–7.5) and declining under alkaline stress. In this study, pH ranged from 8.21 (HB) to 8.67 (XXW), all exceeding the optimal range for bacterial diversity.

XG (calcareous clay-loam; Chao1 5946) and ZH (gravelly sandy loam; Chao1 5710) represent contrasting mechanisms: XG’s higher clay content (36.3%) creates diverse microhabitats with variable water films and oxygen gradients, supporting niche differentiation [48]. ZH’s high gravel content (49.35%) increases physical heterogeneity and aeration, promoting aerobic-facultative metabolic diversity. Both textures enhance diversity through different ecological mechanisms, while intermediate textures (MH: 54.51% gravel, XXW: 87.2% sand) may represent suboptimal conditions.

### 4.2. Bacterial Composition Responses to Soil Properties in Rhizosphere and Bulk Soils

Proteobacteria, Actinobacteriota, and Acidobacteriota are dominant in various soils worldwide [49,50], but their relative abundances vary with texture and management [51,52,53]. Significant composition differences were observed between rhizosphere and bulk soils within the same texture class, contrasting with some previous studies but consistent with texture-mediated rhizosphere effects [45].

All vineyards shared core taxa (Proteobacteria, Actinobacteriota, and Acidobacteriota > 75% cumulative abundance), likely due to common *V. vinifera* root exudate profiles and uniform management (cultivar, training system, and irrigation). However, rare taxa (<1% relative abundance, comprising >40% of observed species-level annotation) exhibited between-site differentiation. However, three caveats can be recognized: (i) rare taxa collectively accounted for <5% of total sequencing reads, limiting their functional contribution; (ii) shotgun metagenomic sequencing depth (6.2–8.7 Gb per sample) may be insufficient for reliable detection of ultra-rare taxa (<0.1% abundance), and their apparent texture associations may reflect stochastic sampling rather than true ecological signals; and (iii) without metatranscriptomic validation, the functional significance of rare taxa remains hypothetical, consistent with the ‘rare biosphere’ debate [54]. Future studies should employ deeper sequencing or single-cell genomics to resolve rare taxa ecology.”

PERMANOVA confirmed that both Texture (R^2^ = 0.28, *p* = 0.001) and Compartment (R^2^ = 0.15, *p* = 0.003) significantly shaped bacterial community structure, while their interaction was non-significant (R^2^ = 0.08, *p* = 0.12; Section 3.3). The non-significant Texture × Compartment interaction indicates that soil texture and rhizosphere/bulk compartment exert additive rather than synergistic effects on bacterial community structure. This suggests that the microbial response to root exudates is qualitatively similar across textures, but the magnitude of texture-driven divergence is comparable in both compartments. This additivity simplifies the interpretation of results, as texture and compartment effects can be considered independently rather than as interacting drivers.

RDA identified TP as the strongest driver of community composition (r^2^ = 0.585, *p* = 0.001), suggesting phosphorus limitation as a key selective pressure in these alkaline, high-pH soils where P bioavailability is reduced by Ca-P precipitation [10]. This is consistent with the unique terroir conditions in the eastern foot of Helan Mountain, where calcareous parent materials dominate. Recent syntheses have highlighted P limitation as a universal driver of soil bacterial community structure in alkaline agricultural systems [55].

The lack of consistent rhizosphere effects on alpha diversity may be attributed to (i) sampling proximity—bulk soil at 50 cm may still be influenced by fine roots (diameter < 2 mm) extending >1 m from the trunk; (ii) drought limitation—arid climate (150–200 mm annual rainfall) may constrain exudate production and diffusion; and (iii) vineyard age—5–8-year-old roots may have established stable microbial associations with reduced exudate novelty. Additionally, uniform management (same fertilizers, pesticides, and irrigation across all sites) may have homogenized microbial communities, masking texture effects. This is indeed a limitation. Future studies should sample bulk soil at >1 m from the trunk and include root exudate metabolomics to quantify rhizosphere gradients. The current design represents a pragmatic compromise for commercial vineyard access but may underestimate true rhizosphere effects.

It is important to note that the 50 cm bulk soil sampling distance likely represents a conservative (i.e., underestimated) assessment of rhizosphere effects. Had bulk soil been sampled at the inter-row center (≥1.75 m from the trunk), the rhizosphere–bulk contrast would likely have been more pronounced, particularly for alpha diversity metrics and rare taxa abundance. This methodological constraint should be considered when interpreting the modest compartment effect observed in PERMANOVA and the relatively high similarity in Shannon diversity between rhizosphere and bulk soils within the same vineyard.

In vineyards with the same texture and different fine-scale composition, such as XG and HB (both calcareous), significant differences in Proteobacteria (53.3% vs. 57.8%), Actinobacteriota (27.1% vs. 16.2%), Acidobacteriota (3.9% vs. 5.0%), and Verrucomicrobiota (1.9% vs. 3.0%) in rhizosphere soil suggest that fine-scale variation in multiple soil properties—including but not limited to clay content, gravel content (9.92% vs. 11.07%), and localized organic matter distribution—may collectively influence water retention and nutrient adsorption. Significant differences in Proteobacteria (53.3% vs. 57.8%), Actinobacteriota (27.1% vs. 16.2%), Acidobacteriota (3.9% vs. 5.0%), and Verrucomicrobiota (1.9% vs. 3.0%) in rhizosphere soil suggest that clay content variation (9.85% vs. 10.79%) alters water retention and nutrient adsorption, selectively favoring different taxa. The observed differences between rhizosphere and bulk soils within the same vineyard likely reflect root-induced changes in soil structure (aggregate formation, organic matter enrichment, and localized nutrient redistribution) rather than inherent particle size variation, given that soil texture is an intrinsic property that is relatively stable at the spatial scale of this study.

### 4.3. Soil Microbial Functions Across Habitats

Notably, this study identified a decoupling between measured enzyme activities and annotated metabolic functions: gravelly soils (ZH and MH) showed highest enzyme activities (ALP, UA, Cat, and IA) but lowest annotated metabolic pathway abundances (AAM, CM, and MCV). For example, R_MH showed high ALP (Figure S2) but low AAM (7.47%) and CM (7.19%) compared to R_XG (AAM 7.84%, CM 7.48%). This hypothesis suggests that enzyme activities reflect extant microbial stress responses and nutrient limitation (higher OM and nutrients in gravelly soils drive higher enzymatic investment), while direct KEGG functional annotation based on DIAMOND alignments reflects genomic potential (more diverse gene repertoires in calcareous and sandy soils due to less selective pressure). This interpretation is speculative and requires validation by metatranscriptomics or proteomics. Additionally, it cannot be excluded that measured enzyme activities reflect, in part, stable extracellular enzymes persisting in the soil matrix (legacy effects) rather than solely current microbial metabolic activity. This underscores the importance of combining metagenomics with enzymatic assays [56,57] and suggests that gravelly soils support metabolically active but functionally narrower communities. Recent integrative studies have demonstrated that enzyme activities reflect extant microbial stress responses, while metagenomic predictions capture genomic potential, with the degree of decoupling varying with soil texture and management intensity [58].

AAM and CM dominated metabolic functions across all habitats, followed by EM and MCV [59], consistent with the presented findings. Rhizosphere soils showed higher metabolic gene abundances than bulk soils for all pathways except EM, likely because root exudates provide labile carbon and nitrogen substrates that stimulate anabolic metabolism (AAM, CM) while EM (respiration) is less substrate-limited and more temperature-dependent.

In different textures, a small proportion of rhizosphere metabolic functions showed significant differences, confirming texture-mediated effects on plant rhizosphere metabolism [60,61]. The higher MTP in R_XG (1.04%) than in other soils (0.93–0.98%) may reflect enhanced terpenoid biosynthesis in calcareous clay-loam rhizospheres, potentially influencing grape berry aromatic compound accumulation. However, this is an annotated function based on gene presence, requiring metatranscriptomic validation to confirm actual expression.

Regarding texture-mediated modulation of uniform management effects, although fertilizers, irrigation, and pesticides were applied uniformly across all vineyards, the extreme textural differences among the sites would have generated divergent leaching and retention dynamics from identical inputs. Aeolian sandy soils (XXW), with high porosity (38%) and minimal clay content (7.37%), favor rapid water infiltration and nutrient leaching, potentially creating nutrient-depleted microsites that select for oligotrophic-adapted taxa. Conversely, calcareous clay-loam soils (XG, HB), with higher clay content (~10%) and lower porosity (40–44%), retain water and nutrients more effectively, sustaining higher metabolic pathway diversity. This indirect driver—texture-mediated differential response to uniform management—likely contributed to the stronger texture effect (PERMANOVA R^2^ = 0.28) than compartment effect (R^2^ = 0.15) and warrants explicit quantification in future studies via lysimeter or soil solution monitoring.

### 4.4. Analysis of Soil Microbial Dynamics

Microbial activities drive carbon and nitrogen sequestration and transformation processes in soils, such as denitrification, nitrification, nitrogen fixation, and anaerobic ammonia oxidation [62,63]. Some microbes in the nitrogen cycle can secrete urease, which is significantly positively correlated with soil nitrogen cycle [64]. UA showed significant positive correlations with TN, AN, TP, and OM (r ≥ 0.4, *p* < 0.01), confirming its role as an indicator of N cycling intensity.

Certain phosphorus-enhancing microbes can secrete soil phosphatase, which could convert insoluble organic phosphorus compounds into available forms and further increase plant phosphorus absorption [62]. ALP correlated strongly with TP, AP, TN, and OM (r ≥ 0.4, *p* < 0.01), supporting the P-limitation hypothesis in these alkaline soils. TP emerged as the strongest RDA driver (r^2^ = 0.585), suggesting that P bioavailability, rather than total P, structures bacterial communities.

Another bacterial group associated with XBM includes Pannonibacter, Zhongshania, Sphingomonas, Plasticicumulans, Nocardioides, Bacillus, and Arthrobacter; these can secrete soil peroxidase, which degrades toxic substances and reduces heavy metal pollution in the soil [22,65,66]. XBM abundance was higher in calcareous and aeolian sandy soils (1.93–1.98%) than in gravelly soils (1.90–1.95%), potentially reflecting lower selection pressure for detoxification in high-OM gravelly soils.

Most of these bacteria are aerobic or strictly aerobic and the rhizosphere soil void was higher than the bulk soils, and therefore the microbial metabolic function of rhizosphere soil was higher than that in the bulk soils. This is consistent with porosity differences: gravelly soils (ZH: 42%, MH: 49%) showed higher aeration than calcareous soils (XG: 44%, HB: 40%) and aeolian sandy soil (XXW: 38%).

In bulk soil, it was observed that the levels of AN, TN, AP, TP, and OM in gravelly soil (ZH and MH) were significantly higher than in other types of bulk soil. Rhizospheric soil also exhibited a similar trend, where AN, TN, AP, TP, and OM significantly influenced soil IA, UA, Cat, and ALP. Consequently, it was observed that the enzyme activities of the four types of enzymes in gravelly soil were significantly higher than in other soil habitats (Figure S2). However, the overall microbial metabolic levels in the gravelly soil habitats were lower than in the calcareous soil and aeolian sandy soil habitats (Table S4), contrary to most studies [67,68]. This paradox may reflect high metabolic investment per unit biomass in gravelly soils (stress response) versus diverse but low-investment metabolic strategies in finer-textured soils (resource conservation).

### 4.5. Study Limitations and Future Directions

This study has several limitations that should be addressed in future research. First, a critical limitation is the hierarchical sampling design: while five vineyards were sampled, the number of independent sites (biological replicates) per texture category was limited (two for gravelly: ZH and MH; two for calcareous: XG and HB; and one for aeolian sandy: XXW). Consequently, the three sampling points within each vineyard represent technical rather than true biological replicates. The limited number of independent vineyards per texture category precluded the application of linear mixed-effects models with adequate statistical power. It is acknowledged that using ANOVA and PERMANOVA without accounting for vineyard-level nesting may inflate Type I error rates and produce false positives; therefore, all cross-site comparisons should be interpreted as exploratory rather than confirmatory. Second, the proximity of bulk soil sampling to the root zone represents a significant methodological constraint. In 5-to-8-year-old vineyards with the described planting spacing (1.0 m × 3.5 m), the grapevine root system extends well beyond 50 cm from the trunk. Previous studies have demonstrated that grapevine fine roots (diameter < 2 mm) and associated mycorrhizal networks can explore soils up to 1 m horizontally from the trunk, with 50% of root length concentrated within 45 cm of the plant row under drip irrigation [69]. Mature grapevine woody roots can extend horizontally up to 10 m from the trunk, and fine roots are predominantly distributed in the top 0.5–1.0 m of soil. Given that bulk soil was sampled only 50 cm from the trunk, it is highly probable that fine roots and root exudates still influenced these samples, thereby attenuating the true magnitude of the rhizosphere effect. This extreme proximity likely contributed to the observed similarity in alpha diversity between rhizosphere and bulk soils (Shannon index: 5.77–5.95 vs. 5.24–5.69), potentially masking stronger rhizosphere-driven differentiation. The lack of consistent rhizosphere effects on alpha diversity and the relatively modest compartment effect in PERMANOVA (R^2^ = 0.15, *p* = 0.003) compared to texture effects (R^2^ = 0.28, *p* = 0.001) may partially reflect this sampling artifact. True bulk soil in commercial vineyards is conventionally sampled at the center of the inter-row (approximately 1.75 m from the trunk in the 3.5 m row spacing), where root influence is minimal. Future studies should employ distances > 1 m from the trunk or use isotope tracing to definitively exclude root-derived carbon inputs and capture the full spectrum of rhizosphere–bulk soil gradients. Third, the classification of “soil textures” versus “soil types” requires refinement: although USDA texture classes and gravel content were used, additional pedogenic characterization (mineralogy, soil structure stability, and aggregate size distribution) would strengthen causal inference. Fourth, while vineyard management was uniform, the soil solution chemistry or leachate nutrients were not monitored to quantify how texture-specific hydrological properties modulated the microbial response to identical agronomic inputs. Fifth, uniform vineyard management (fertilizers, pesticides, and irrigation) across sites may have homogenized communities, confounding texture effects; a manipulative experiment controlling for management would isolate texture effects. Sixth, annotated metabolic functions require validation by metatranscriptomics or proteomics to confirm actual gene expression. Finally, seasonal sampling (dormancy, flowering, and harvest) would capture temporal dynamics absent in this single veraison sampling.

## 5. Conclusions

This study provides novel insights into soil texture-mediated bacterial community assembly in vineyard ecosystems. Four key conclusions emerge:

(1) Fine-scale physical heterogeneity within broad pedogenic classes is a stronger predictor of bacterial community structure than categorical soil classification alone. Calcareous soils with different clay contents (XG: 9.85% clay, 9.92% gravel vs. HB: 10.79% clay, 11.07% gravel) showed greater community divergence than soils of different pedogenic origin but similar texture, suggesting that continuous texture gradients rather than discrete taxonomic classes drive microbial differentiation. However, given the limited number of independent sites per texture category and the presence of uncontrolled confounding factors (e.g., slope, microclimate, and localized organic matter), causal attribution to specific particle size fractions is not warranted. This challenges the traditional emphasis on soil taxonomic classification in terroir studies and highlights the need for fine-scale physical characterization in vineyard management.
(2) The rhizosphere homogenizes bacterial alpha diversity, but not community composition, across textures. Root exudates likely provided a common carbon source that equalized diversity metrics (Shannon index: 5.77–5.95 across rhizosphere soils), while texture-dependent nutrient availability (particularly P, N, and OM) maintained compositional differences (PERMANOVA, Texture R^2^ = 0.28, *p* = 0.001). However, the modest magnitude of the compartment effect (R^2^ = 0.15) and the relatively small rhizosphere–bulk divergence in alpha diversity may have been attenuated by the 50 cm bulk soil sampling distance, which likely fell within the zone of fine root and mycorrhizal influence in these 5-to-8-year-old vines. True bulk soil at the inter-row center would likely reveal a stronger rhizosphere effect. The lack of consistent rhizosphere effects in some comparisons reflects sampling proximity limitations (50 cm bulk soil) and uniform management across sites.
(3) Phosphorus and organic matter were the primary community drivers, with total phosphorus (TP) showing the strongest effect. TP (r^2^ = 0.585, *p* = 0.001), OM (r^2^ = 0.518, *p* = 0.001), and AN (r^2^ = 0.467, *p* = 0.001) emerged as the strongest RDA constraints, highlighting P limitation as a key ecological filter in alkaline vineyard soils where Ca-P precipitation reduces bioavailability. This finding is consistent with the unique terroir conditions in the eastern foot of Helan Mountain.
(4) Annotated metabolic potential decouples from measured enzyme activity. Gravelly soils showed highest enzyme activities (ALP, UA, Cat, and IA in ZH and MH: Figure S2) but lowest annotated pathway diversity (AAM: 7.47–7.60% vs. 7.72–7.84% in calcareous soils), suggesting texture-induced stress selects for functionally specialized but taxonomically constrained communities. This disconnect underscores that KEGG-based functional predictions from shotgun metagenomic data represent genomic potential. These findings refine the concept of “terroir” by demonstrating that texture-scale heterogeneity (operating at cm to m scales) is a critical, previously underappreciated driver of vineyard microbial biodiversity [47,70,71]. This aligns with emerging precision viticulture frameworks that advocate for site-specific soil management to optimize grape quality and ecosystem sustainability [72,73]. These findings generate testable hypotheses for future precision viticulture research: (i) whether targeted organic matter amendments in coarse textures can enhance metabolic diversity; (ii) whether phosphorus biofortification in calcareous soils can relieve P limitation; and (iii) whether gravel content optimization can balance aeration and water retention for microbial activity. These hypotheses require validation through manipulative experiments before implementation in vineyard management.

## Figures and Tables

**Table 1:** Alpha diversity index characteristics of bacterial communities among different soil types.

Sample	Chao1	ACE	Shannon	Simpson	Coverage
R_MH	5993 ± 151 bc	6012 ± 176 bc	5.85 ± 0.02 ab	0.0101 ± 0.0002 de	99.0%
R_XXW	5987 ± 91 bc	6004 ± 165 bc	5.82 ± 0.08 abc	0.0101 ± 0.0011 de	99.0%
R_ZH	5710 ± 137 c	5725 ± 145 c	5.77 ± 0.07 bc	0.0100 ± 0.0009 de	99.0%
R_XG	5946 ± 112 bc	5944 ± 110 bc	5.95 ± 0.03 a	0.0084 ± 0.0009 e	99.0%
R_HB	6517 ± 245 a	6534 ± 229 a	5.94 ± 0.12 a	0.0088 ± 0.0012 e	98.9%
NR_MH	5868 ± 206 bc	5967 ± 167 bc	5.54 ± 0.04 de	0.0168 ± 0.0008 bc	99.0%
NR_XXW	5849 ± 475 bc	5940 ± 479 bc	5.58 ± 0.10 de	0.0143 ± 0.0017 cd	99.0%
NR_ZH	5196 ± 271 d	5266 ± 280 d	5.24 ± 0.12 f	0.0228 ± 0.0041 a	99.1%
NR_XG	5686 ± 199 c	5698 ± 224 c	5.44 ± 0.14 e	0.0190 ± 0.0057 ab	99.0%
NR_HB	6284 ± 285 ab	6351 ± 256 ab	5.69 ± 0.08 cd	0.0138 ± 0.0019 cd	98.9%

Different lowercase letters indicate significant differences at *p* < 0.05.

## Data Availability

The original contributions presented in this study are included in the article/Supplementary Materials. Further inquiries can be directed to the corresponding authors.

## Supplementary Materials

The following supporting information can be downloaded at: https://www.mdpi.com/article/10.3390/microorganisms14071504/s1, Figure S1. Rarefaction curves of ofbacteria. NR_HB: non-rhizosphere soils in the Hongbao Wine-growing base; NR_MH: non-rhizosphere soils in the Meihe Manor; NR_XG non- rhizosphere soils in the Xige Winery; NR_XXW: non-rhizosphere soils in the: Xixia Wang Winery; NR_ZH: non-rhizosphere soils in the: Zhi Hui Yuanshi Chateau; R_HB: rhizosphere soils in the Hongbao Wine-growing base; R_MH: rhizosphere soils in the Meihe Manor; R_XG: rhizosphere soils in the Xige Winery; R_XXW: rhizosphere soils in the: Xixia Wang Winery; R_ZH: rhizosphere soils in the: Zhi Hui Yuanshi Chateau; Figure S2. Physicochemical properties of rhizosphere and non-rhizosphere soils in various habitats. OM: the concentrations of Soil organic matter: pH: soil pH; EC: soil Electrical conductivity; AN: soil alkali-hydrolyzed nitrogen concentration; TN: soil total nitrogen concentration; AP: soil available phosphorus content; TP, soil total phosphorus concentration; AK soil available K concentration; Ca: Soil ca concentration; Mg: soil Mg concentration; Al: soil Al concentration; Fe: Fe concentration in soil; ALP, soil alkaline phosphatase activity; UA: Urease activity in soil; Cat: catalase activity in soil; IA: Soil Invertase activity. Different lowercase letters meant significant differences among different sample groups at 0.05 level Table S1. Vineyard planting information and soil information; Table S2. Core microbial community, top 1% of bacteria at the phylum level; Table S3. Significance of the effect of soil variables on microbial community composition in different soil habitats; Table S4. Functional analysis of the inter- and non-root microbial communities of Cabernet Sauvignon grapes in different habitats. Table S5. LEfSe_Biomarkers.xlsx.
