# Supplementary material for: Bacterial Diversity, Structure, and Function in Rhizosphere and Bulk Soils of Grapevines: Comparing Gravelly, Calcareous, and Aeolian Sandy Textures
Source: Microorganisms. 2026 Jul 9;14(7):1504. doi: 10.3390/microorganisms14071504 (PMC13414349; doi:10.3390/microorganisms14071504)
Supplement: Supplementary file 1 [file microorganisms-14-01504-s001.zip › Supplementary Material.pdf]

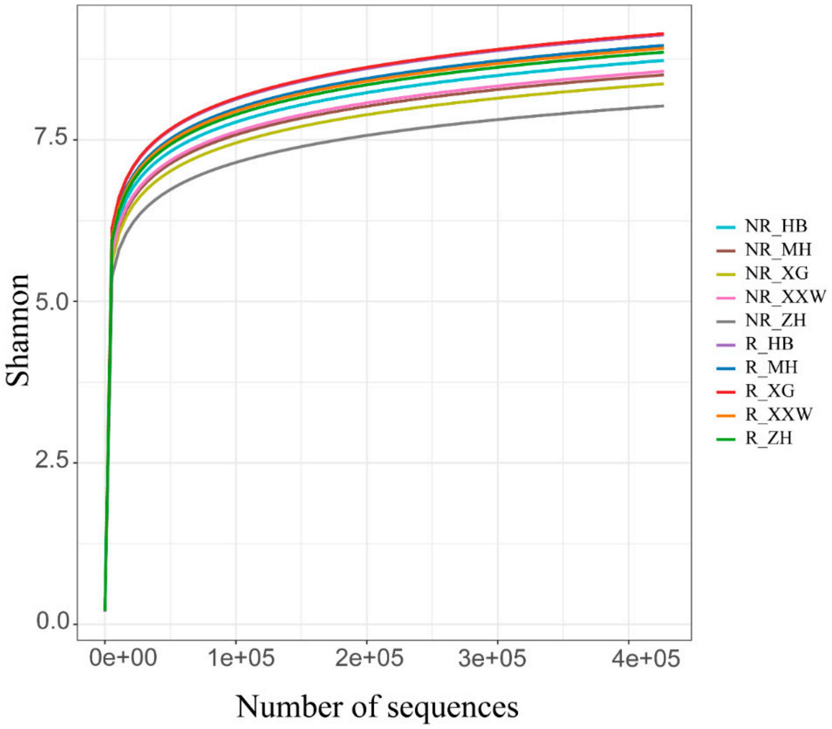

Fig. S1. Rarefaction curves of ofbacteria. NR_HB : non-rhizosphere soils in the Hongbao Wine-growing base; NR_MH: non- rhizosphere soils in the Meihe Manor;NR_XG non- rhizosphere soils in the Xige Winery;NR_XXW: non- rhizosphere soils in the:Xixia Wang Winery;NR_ZH: non-rhizosphere soils in the: Zhi Hui Yuanshi Chateau； R_HB : rhizosphere soils in the Hongbao Wine-growing base; R_MH: rhizosphere soils in the Meihe Manor;R_XG： rhizosphere soils in the Xige Winery;R_XXW: rhizosphere soils in the:Xixia Wang Winery;R_ZH: rhizosphere soils in the: Zhi Hui Yuanshi Chateau；

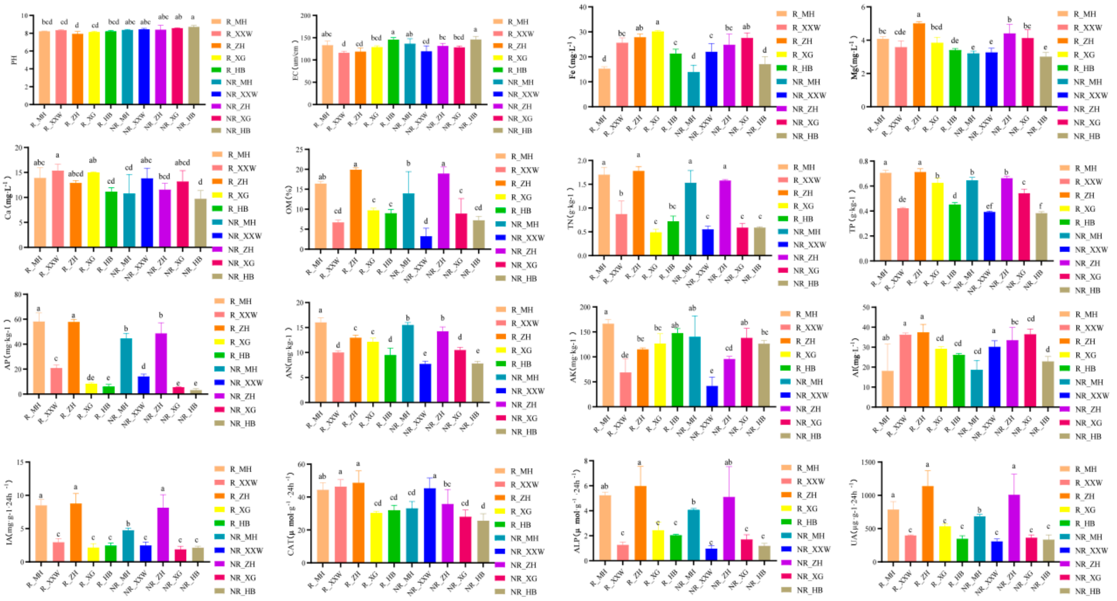

Fig. S2. Physicochemical properties of rhizosphere and non-rhizosphere soils in various habitats. OM: the concentrations of Soil organic matter; pH: soil pH;EC: soil Electrical conductivity;AN: soil alkali-hydrolyzed nitrogen concentration;TN: soil total nitrogen concentration;AP: soil available phosphorus content;TP, soil total phosphorus concentration;AK soil available K concentration;Ca: Soil ca concentration;Mg: soil Mg concentration;Al: soil Al concentration;Fe: Fe concentration in soil;ALP, soil alkaline phosphatase activity;UA: Urease activity in soil;Cat: catalase activity in soil;IA: Soil Invertase activity

Different lowercase letters meant significant differences among different sample groups at 0.05 level.

Table S1 Vineyard planting information and soil information

Vineyard	Soil type	Planting spacing	Planting direction	Gravel (%)	Sand (%)	Silt (%)	Clay (%)	Bulk density (g.)	specific gravity	Porosity (%)
Zhihuiyuanshi	Gravelly Soil	1*3.5	North-south	49.35	47.16	2.04	1.45	1.51	2.68	42
Meihe	Gravelly Soil	1*3.5	North-south	54.51	41.48	2.56	1.46	1.38	2.7	49
Xixia Wang	Aeolian Sandy Soil	1*3.5	North-south	3.87	79.50	9.26	7.37	1.68	2.7	38
Xige	Calcareous Soil	1*3.5	North-south	9.92	66.55	13.69	9.85	1.45	2.52	44
Hongbao	Calcareous Soil	1*3.5	North-south	11.07	62.76	15.38	10.79	1.44	2.41	40

Table S2 Core microbial community, top 1% of bacteria at the phylum level

phylum	R_MH%	R_XXW%	R_ZH%	R_XG%	R_HB%	NR_MH%	NR_XXW%	NR_ZH%	NR_XG%	NR_HB%	R%	NR%	Total%
Proteobacteria	47.7±1bcd	54.3±1.2ab	56.1±2a	53.3±3.1abc	57.8±1.8a	44.3±4.1d	42.8±2.5d	31.8±3.4e	42.6±9.8d	46.4±0.6cd	53.9****	41.6	47.7A
Actinobacteriota	29.4±1.1a	14.8±0.5c	18.6±2.5bc	27.1±4a	16.2±1.2c	24.5±5.9ab	20.3±2.2bc	30.1±3.6a	28.7±7.9a	19.3±2.2bc	21.2	24.6	22.9B
Acidobacteriota	3.5±0.6d	6.9±0.4b	5.1±0.8c	3.9±1.4cd	5±1.1bcd	6.6±0.6b	9.2±0.9a	9.5±0.7a	7.3±1.1b	7.6±0.2b	4.9	8****	6.5C
Chloroflexota	3.2±0.2ef	3.8±0.6de	4.3±0.4cde	2.1±0.5f	3.2±0.2ef	6±1.5ab	6.4±0.6ab	6.9±0.3a	4.6±0.9cd	5.4±0.4bc	4.4	5.9****	4.6D
Bacteroidota	4.5±0.8ab	3.7±0.3abcd	3.9±0.5abc	5.4±1.1a	4.6±0.3ab	1.8±0.7cde	2.5±2.5bcde	1.3±0.3e	1.6±0.9de	4.6±1.9ab	3.3***	3.1	3.4E
Gemmatimonadota	2.3±0.3bcd	2.3±0.1bcd	2.2±0.3bcd	1.7±1d	2.1±0.5cd	3±0.1abc	3±0.4ab	3.6±0.4a	3.2±0.6a	2.8±0.3abc	2.9****	2.8	2.6F
Verrucomicrobiota	3.4±0.6ab	3.8±0.3a	2.1±0.5cedf	1.9±0.3def	3±0.5abc	1.8±0.4def	2.4±0.6cde	1.3±0.2f	1.7±0.6ef	2.7±0.7bcd	2.1	2.7**	2.4FG
Methylomirabilota	1.5±0.1cd	2.4±0.4bd	1.6±0.5bcd	1±0.5d	1.7±0.5bcd	2.3±0.9bcd	2.9±0.7b	5.1±1a	2.6±1.1bc	1.5±0.4cd	1.6	2.4**	2.2FG
Latescibacterota	1.3±0de	1.5±0.1d	1.6±0.1d	0.9±0.3e	1.1±0.2de	3±0.2ab	2.7±0.3b	3.2±0.4a	2.1±0c	2.7±0.5ab	1.4	2****	2.0 G
SAR324	0.8±0.1de	2.2±0.4a	1.2±0.1cd	0.7±0.3e	2±0.2ab	1.4±0.1c	2.2±0.3a	2.3±0.3a	2±0.3ab	1.6±0.1bc	1.3	1.9****	1.6 H’
Planctomycetota	0.8±0ef	1±0.1de	0.8±0.2ef	0.7±0.1f	1.2±0.3cd	1.7±0.3ab	1.8±0.1a	1.4±0.1bc	1.2±0cd	2±0.2a	0.9	1.6***	1.2 I
Myxococcota	0.6±0f	1.2±0.2bc	1±0.2cde	0.7±0.1ef	0.9±0.2cdef	1.6±0.3a	1.6±0.2a	0.8±0.1def	1.1±0.2bcd	1.4±0.1ab	0.9	1.3***	1.1 I
Nitrospirota	0.4±0.1c	1.5±0.9a	0.9±0.2abc	0.3±0.1c	0.6±0.2bc	1±0.3abc	0.9±0abc	1.2±0.5ab	0.6±0bc	0.9±0.1abc	0.8	0.9	0.8J

Different lowercase letters in each column meant significant differences among different sample groups at 0.05 level.. “*”The significant level of difference between the R combination NR groups is 0.05, “* * ”represents the level of difference is 0.01, “* * *” represents the level of difference is 0.001, and “* * * *” represents the level of difference is 0.0001. The significant level of difference between groups in the capitalization alphabet is 0.05

Table S3 Significance of the effect of soil variables on microbial community composition in different soil habitats.

	pH	EC	AN	TN	AP	TP	AK	OM	ALP	IA	UA	cat	Ca	Mg	Al	Fe
RDA1	0.877	0.981	0.114	0.294	0.197	-0.046	-0.44	0.197	0.083	0.163	0.171	-0.862	-0.792	0.057	0.658	-0.27
RDA2	0.481	-0.192	-0.994	-0.956	-0.98	-0.999	-0.898	-0.98	-0.997	-0.987	-0.985	0.507	-0.611	-0.998	-0.753	-0.963
r2	0.378	0.014	0.467	0.235	0.215	0.585	0.319	0.518	0.268	0.217	0.27	0.032	0.123	0.399	0.035	0.091
Pr(>r)	0.002**	0.823	0.001***	0.026*	0.036*	0.001***	0.009**	0.001***	0.019*	0.032*	0.016*	0.618	0.156	0.001***	0.603	0.286

OM: the concentrations of Soil organic matter； pH: soil pH;EC: soil Electrical conductivity;AN: soil alkali-hydrolyzed nitrogen concentration;TN: soil total nitrogen concentration;AP: soil available phosphorus content;TP, soil total phosphorus concentration;AK soil available K concentration;Ca： Soil ca concentration;Mg: soil Mg concentration;Al: soil Al concentration;Fe: Fe concentration in soil;ALP, soil alkaline phosphatase activity;UA: Urease activity in soil;Cat: catalase activity in soil;IA: Soil Invertase activity， Significant effect: * $p < 0.05$, ** $p < 0.01$. *** $p < 0.001$

Table S4 Functional analysis of the inter- and non-root microbial communities of Cabernet Sauvignon grapes in different habitats

Sample	R_MH%	R_XXW%	R_ZH%	R_XG%	R_HB%	NR_MH%	NR_XXW%	NR_ZH%	NR_XG%	NR_HB%	R%	NR	Total
AAM	7.47±0.1bcd	7.6±0.12ab	7.58±0.02abc	7.84±0.09a	7.72±0.15ab	6.88±0.29e	7.4±0.15bcd	6.62±0.33d	7.25±0.23cd	7.19±0.1d	7.64	7.07	7.35A
CM	7.19±0.09ab	7.28±0.02ab	7.23±0.04ab	7.48±0.11a	7.21±0.07ab	6.69±0.24de	7.24±0.17ab	6.47±0.31e	7.13±0.19bc	6.89±0.15cd	7.28	6.88	7.08B
EM	6.23±0.06d	6.64±0.15bc	6.57±0.08bc	6.27±0.12d	6.46±0.05cd	6.44±0.21cd	6.94±0.12a	6.24±0.2d	6.56±0.22bc	6.79±0.05ab	6.43	6.59	6.51C
MCV	3.28±0.06a	3.27±0.09a	3.2±0.06abc	3.36±0.11a	3.33±0.03a	2.93±0.19de	3.06±0.03bcd	2.81±0.13e	3.02±0.09cd	3.24±0.13ab	3.29	3.01	3.15D
NM	2.61±0.03abc	2.63±0.03abc	2.62±0.04abc	2.72±0.03a	2.67±0.03ab	2.52±0.13c	2.68±0.01ab	2.33±0.1d	2.58±0.09bc	2.65±0.05ab	2.65	2.55	2.6E
UM	2.03±0.05a	2.02±0.09a	1.96±0.1a	2.01±0.1a	1.99±0.03b	1.68±0.04b	1.7±0.04b	1.72±0.08b	1.74±0.03b	1.71±0.03b	2	1.71	1.85F
LM	1.99±0.04ab	1.99±0.05ab	1.94±0.04b	2.08±0.08a	2.04±0.06ab	1.67±0.07de	1.73±0.03cd	1.58±0.07e	1.8±0.09c	1.65±0.08de	2.01	1.69	1.85F
XBM	1.95±0.04a	1.93±0.02a	1.9±0.05ab	1.98±0.07a	1.91±0.08a	1.57±0.1d	1.65±0.06cd	1.67±0.04c	1.8±0.05b	1.55±0.03d	1.93	1.65	1.79F
MOAA	1.72±0.01b	1.71±0.03b	1.7±0.04b	1.84±0.05a	1.73±0.03b	1.46±0.05d	1.56±0.04c	1.43±0.08d	1.57±0.05c	1.45±0.03d	1.74	1.49	1.62G
BOSM	1.2±0.01a	1.18±0.01ab	1.18±0.01ab	1.2±0.05a	1.16±0.02ab	1.06±0.06cd	1.13±0.03bc	1.03±0.07d	1.12±0.04bc	1.13±0.02bc	1.18	1.09	1.14H
MTP	0.98±0.02b	0.94±0.03b	0.93±0.01b	1.04±0.04a	0.96±0.01b	0.85±0.07c	0.87±0.03c	0.85±0.03c	0.93±0.01b	0.84±0.02c	0.97	0.87	0.92I
GBM	0.93±0.04a	0.88±0.04ab	0.84±0.03b	0.96±0.06a	0.89±0.03ab	0.69±0.04cd	0.72±0.04c	0.63±0.05d	0.71±0.03cd	0.7±0.07cd	0.9	0.69	0.8J

AAM： Amino acid metabolism； CM： Carbohydrate metabolism； EM: Energy metabolism; MCV： Metabolism of cofactors and vitamins； NM： Nucleotide metabolism； UM： Unclassified: metabolism； LM： Lipid metabolism； XBM： Xenobiotics biodegradation and metabolism； MOAA: Metabolism of other amino acids; BOSM： Biosynthesis of other secondary metabolites； MTP: Metabolism of terpenoids and polyketides; GBM: Glycan biosynthesis and metabolism.,Different lowercase letters in each column meant significant differences among different sample groups at 0.05 level.. The significant level of difference between groups in the capitalization alphabet is 0.05.
